# Colonic Pseudolipomatosis: A Spectrum of Two Illustrated Cases of an Uncommon Condition

**DOI:** 10.7759/cureus.81103

**Published:** 2025-03-24

**Authors:** Ygor R Fernandes, Mateus P Funari, Alex R Fonseca, Francisco S Koyama, Eduardo Guimarães de Moura

**Affiliations:** 1 Gastroenterology, Hospital das Clínicas da Faculdade de Medicina da Universidade de São Paulo (HCFMUSP), São Paulo, BRA; 2 Gastroenterology, A.C. Camargo Cancer Center, São Paulo, BRA; 3 Graduate Program in Sciences in Gastroenterology, Faculdade de Medicina da Universidade de São Paulo (FMUSP), São Paulo, BRA

**Keywords:** benign colonic lesions, colonic pseudolipomatosis, colonoscopic findings, differential diagnosis in colonoscopy, disinfection, histopathology

## Abstract

Colonic pseudolipomatosis (CP) is a rare, benign condition typically identified incidentally during routine colonoscopies. Characterized by gas-filled vacuoles within the colonic mucosa, CP is often asymptomatic and self-limiting but can be misinterpreted as more serious conditions, such as ischemic colitis or pseudomembranous colitis. We report two cases of CP with distinct clinical presentations: a 55-year-old asymptomatic male patient with an incidental lesion found during routine screening and a 73-year-old male who developed lower gastrointestinal bleeding 10 days after an extended colonoscopy with multiple polypectomies. Histopathological analysis confirmed CP in both cases. The first patient required no further intervention, while the second underwent supportive care and a follow-up colonoscopy, which showed resolution of the lesions. These cases highlight the spectrum of CP, emphasizing the importance of recognizing this entity to avoid misdiagnosis and unnecessary interventions. Furthermore, they underscore the role of careful endoscopic techniques and disinfection protocols in minimizing the occurrence of iatrogenic CP.

## Introduction

Colonic pseudolipomatosis (CP) is an uncommon, benign condition of the colon, often detected incidentally during routine colonoscopies. Histopathologically, CP is characterized by gas-filled vacuoles within the lamina propria, which appear as optically empty spaces under microscopy. Its reported incidence is low, ranging from 0.01% to 0.3% of colonoscopies [1]. Although generally asymptomatic and self-limiting, CP can be misinterpreted as more concerning conditions, such as ischemic colitis, pseudomembranous colitis, lipomas, or lymphangiomas, leading to potential diagnostic challenges [2].

The etiology of CP remains debated, with both mechanical and chemical factors implicated in its development. Overinflation during endoscopy and exposure to disinfectants, particularly hydrogen peroxide, are suspected triggers. Improper rinsing of endoscopic channels has also been suggested as a contributing factor, reinforcing the importance of rigorous disinfection protocols [2,3].

The diagnosis of CP is established through endoscopic appearance and histopathological confirmation [3]. Endoscopically, CP presents as whitish, slightly elevated mucosal lesions, often without ulceration or bleeding. A biopsy is essential to differentiate CP from other mucosal lesions, as histology confirms the presence of gas-filled vacuoles without dysplasia or malignancy [3]. Given its benign nature, treatment is generally unnecessary, with most cases resolving spontaneously. However, awareness among endoscopists is crucial to prevent misdiagnosis and unnecessary interventions [4].

Although CP is typically benign, rare cases may present with complications [4]. In this report, we describe two cases of CP with distinct clinical courses: one detected incidentally in an asymptomatic patient and another associated with post-colonoscopy lower gastrointestinal bleeding. These cases highlight the clinical spectrum of CP and underscore the need for careful procedural techniques to minimize iatrogenic occurrences.

## Case presentation

First case

A 55-year-old male patient with no significant medical history underwent a routine screening colonoscopy for colorectal cancer (CRC) prevention. The patient had no gastrointestinal symptoms, a history of previous colonoscopies, or known exposure to disinfectants associated with CP development. He was not on anticoagulants or any medications known to affect mucosal integrity.

**Figure 1:**
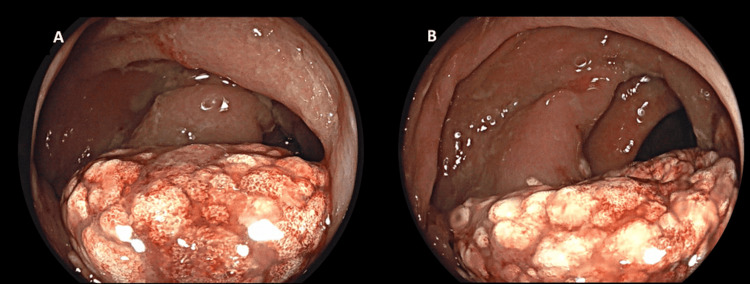
Colonoscopic images (A, B) depicting an elevated, edematous, and whitish lesion in the ascending colon.

During the colonoscopy, a large, elevated, edematous, and whitish lesion measuring approximately 5 cm was identified in the proximal ascending colon, occupying nearly 50% of the lumen. The lesion showed no ulceration or bleeding (Figure 1). A biopsy was performed to rule out malignancy, and the procedure was completed without complications.

**Figure 2:**
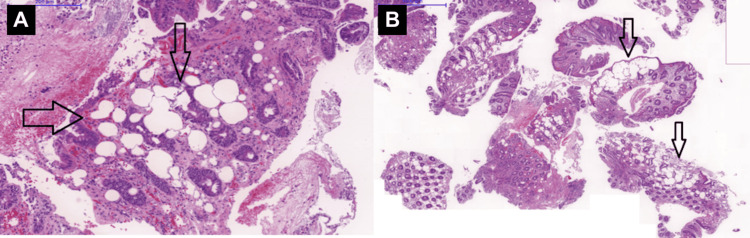
Histopathological analysis of the biopsy. (A) H&E, 200×: Multiple large, optically clear vacuoles (arrows) within the lamina propria, surrounded by mild inflammation; (B) Right, H&E 100×: numerous gas-filled vacuoles (arrows) within the glandular architecture, consistent with the benign nature of colonic pseudolipomatosis. H&E: hematoxylin and eosin

Histopathological analysis revealed multiple variably sized, optically empty vacuoles within the lamina propria, surrounded by a mild inflammatory infiltrate. There was no evidence of glandular destruction, dysplasia, or malignancy, confirming the diagnosis of CP (Figure 2). Immunohistochemical analysis was not required, given the clear histological findings.

The patient was informed of the benign nature of CP and was advised that no specific treatment was required. However, given the lack of long-term studies on CP and the need to ensure symptom resolution, a 30-day follow-up was scheduled. During this period, the patient remained completely asymptomatic, with no gastrointestinal complaints or signs of delayed complications. Considering CP's self-limiting nature and the absence of persistent symptoms, no further follow-up was deemed necessary, and the patient was advised to continue routine CRC screening per standard guidelines.

Second case

A 73-year-old male patient with a history of depression underwent a screening colonoscopy, which revealed pan-diverticular disease, 10 sessile polyps, and three laterally spreading tumors (LSTs), primarily located in the transverse colon. The lesions ranged in size from 2 mm to 35 mm and were removed using forceps polypectomy, snare polypectomy, or mucosectomy. The largest lesion (35 mm in the descending colon) was scheduled for a later endoscopic submucosal dissection (ESD). The total procedure lasted 75 minutes due to the number of interventions performed.

Ten days later, the patient presented with hematochezia. Initial laboratory tests showed hemoglobin levels of 13.2 g/dL (normal range: 13-17 g/dL) and hematocrit of 38.8% (normal range: 40-50%). A follow-up colonoscopy was performed to determine the source of bleeding. The previously resected polyp sites were completely healed, with no visible ulcerations, erosions, or active bleeding, effectively ruling out post-polypectomy hemorrhage as the cause. Additionally, no diverticular bleeding or other sources of hemorrhage were identified.

**Figure 3:**
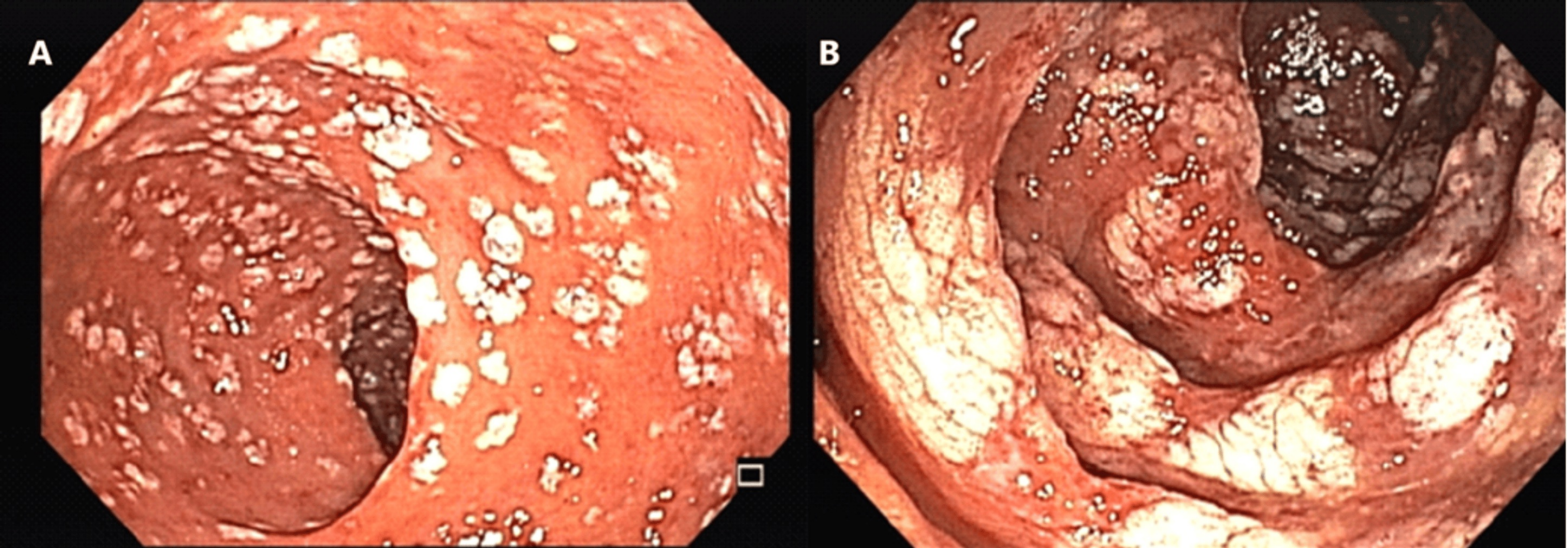
Colonoscopic images (A, B) showing colonic pseudolipomatosis.

However, endoscopic evaluation revealed intense and diffuse erythema, particularly in the cecum and ascending colon, associated with slightly elevated, oval-shaped, and occasionally confluent whitish lesions (Figure 3). These lesions resembled those previously described in CP. Biopsies confirmed acute colitis with polymorphonuclear infiltration in the lamina propria, cryptitis, and cystic spaces within the mucosa, consistent with CP. Given the absence of alternative bleeding sources and the diffuse nature of the CP lesions, the bleeding episode was attributed to CP-related mucosal fragility. The patient received supportive care, and the bleeding resolved without further intervention.

Two months later, the patient underwent ESD for the previously scheduled LST in the descending colon, which had been initially deferred due to its size and the need for an advanced resection technique. The histopathological examination of the resected lesion confirmed a tubulovillous adenoma with low-grade dysplasia. During this procedure, a repeat colonoscopy demonstrated complete resolution of the pseudolipomatous lesions, further supporting the diagnosis of CP as a transient condition. No additional episodes of gastrointestinal bleeding were reported during follow-up.

## Discussion

CP is a rare, benign, and often asymptomatic condition, usually detected incidentally during colonoscopy [1,2]. Histologically, CP is characterized by gas-filled vacuoles within the lamina propria, which appear as optically empty spaces under microscopy [3]. Although CP is self-limiting, its endoscopic appearance can mimic more concerning conditions, necessitating histopathological evaluation to confirm the diagnosis and avoid unnecessary interventions [2,4]. The pathogenesis of CP remains debated, but current evidence suggests an iatrogenic origin, with both mechanical and chemical factors playing a role [5,6]. Excessive insufflation pressure and exposure to disinfectants, particularly hydrogen peroxide, have been implicated as potential triggers leading to gas entrapment within the mucosa [6,7]. Recognizing these contributing factors is crucial in reducing the incidence of CP and ensuring accurate diagnosis [4,8].

The two cases presented in this report illustrate the clinical spectrum of CP, ranging from an asymptomatic incidental finding to a rare presentation with gastrointestinal bleeding. The first case represents the typical course of CP, detected in an asymptomatic patient undergoing routine CRC screening. The lesion was identified as a slightly elevated, whitish mucosal alteration, which prompted a biopsy to rule out neoplastic or inflammatory conditions [1,5]. Histopathological examination confirmed the presence of gas-filled vacuoles with no signs of dysplasia, malignancy, or endothelial-lined spaces, consistent with CP [3,6]. The patient remained asymptomatic during a 30-day follow-up, reinforcing the benign nature of CP and its tendency to resolve spontaneously [2,9]. As CP does not typically progress or cause complications, routine surveillance is not required, and follow-up should be considered only in symptomatic cases or when diagnostic uncertainty persists [4].

The second case presented an atypical scenario in which the patient developed lower gastrointestinal bleeding 10 days after undergoing an extended colonoscopy with multiple polypectomies. While CP is not classically associated with bleeding, the endoscopic evaluation revealed intense and diffuse erythema in the cecum and ascending colon, along with multiple slightly elevated, oval, and occasionally confluent whitish lesions [3,7]. The histopathological findings were consistent with CP, and no alternative sources of bleeding were identified, as the previously resected polyp sites were completely healed without ulceration, erosions, or active hemorrhage [5,8]. The absence of diverticular bleeding or vascular abnormalities further supported CP as the most plausible explanation for the bleeding episode [6,9]. Although the association between CP and gastrointestinal bleeding is not well established, the extensive mucosal involvement observed in this patient raises the possibility that, in rare cases, CP-related lesions may contribute to mucosal fragility and subsequent hemorrhage [4,10]. However, given the lack of definitive evidence linking CP directly to bleeding, further studies are required to explore this potential complication [1,7]. The patient's symptoms resolved with supportive care, and a follow-up colonoscopy two months later demonstrated complete resolution of the pseudolipomatous lesions, reinforcing the transient nature of CP [5,6].

Differentiating CP from other histopathological entities is essential to ensure accurate diagnosis and prevent unnecessary interventions. Several conditions can mimic CP, including lipomas, lymphangiomas, pseudomembranous colitis, and ischemic colitis [8]. Lipomas are characterized by true adipose tissue, which is absent in CP, whereas lymphangiomas contain endothelial-lined cystic spaces, which were not observed in our cases [6,10]. Pseudomembranous colitis typically presents with fibrinous exudates and neutrophilic infiltration, findings that were not identified in our histological evaluation [3,7]. Ischemic colitis is another important differential diagnosis, as it often exhibits coagulative necrosis and capillary thrombi, neither of which were present in our cases [5,9]. The histopathological confirmation of CP, without evidence of fat cells, endothelial proliferation, necrotic tissue, or inflammatory membrane formation, allowed for the exclusion of these alternative diagnoses [1,4]. This reinforces the importance of biopsy in cases where CP is suspected, particularly when the endoscopic findings raise concerns for more serious pathology [6,10].

The follow-up strategies for CP remain undefined, as most cases resolve spontaneously without intervention [2,5]. In the first case, a 30-day follow-up was conducted to confirm the absence of symptoms and ensure spontaneous resolution [3,9]. The literature suggests that CP lesions typically regress within weeks to months, and long-term follow-up is not warranted unless the patient develops new symptoms [7,8]. In the second case, the follow-up was extended due to the bleeding episode, and a repeat colonoscopy demonstrated complete resolution of CP lesions [4,10]. This case highlights that, while CP generally follows a benign course, extensive involvement may result in transient mucosal fragility, requiring short-term observation in symptomatic cases [6,9]. Nevertheless, the role of CP in gastrointestinal bleeding remains unclear, and further investigations are needed to establish whether CP can independently cause hemorrhagic complications or if its presence is coincidental in such cases [2,7].

From an endoscopic perspective, awareness of CP is essential to prevent misdiagnosis and unnecessary interventions [5,10]. Because CP does not require treatment, recognizing its endoscopic features can reduce unnecessary biopsies, avoid repeat procedures, and prevent patient anxiety [1,3]. Furthermore, given that CP is believed to be iatrogenic, minimizing contributing factors such as prolonged procedure times, excessive insufflation pressures, and inadequate rinsing of disinfectants could help reduce its occurrence [6,8]. In clinical practice, careful endoscopic technique and strict adherence to disinfection protocols may play a role in preventing CP, particularly in high-risk cases where extensive endoscopic procedures are required [7,9]. While CP remains a rare and benign entity, its potential to mimic more serious conditions and, in rare cases, to present with symptoms underscores the importance of proper diagnosis and clinical awareness [4,10].

Recognizing CP is crucial to avoid misdiagnosis and unnecessary interventions, and optimizing endoscopic techniques and disinfection protocols may help minimize its occurrence [2,5,9]. Our report highlights the spectrum of CP's clinical presentations and reinforces the need for continued research into its pathogenesis, natural history, and potential clinical implications [1,3,7].

## Conclusions

In summary, CP is a rare but benign condition that typically follows an asymptomatic course, as demonstrated in our first case. However, as illustrated by the second case, CP can, in rare instances, present with complications such as gastrointestinal bleeding when the lesions are extensive. This atypical presentation emphasizes the need for clinicians to consider CP as a potential source of post-colonoscopic complications, even when polypectomy sites are not implicated. Biopsy remains crucial for distinguishing CP from more serious conditions such as colitis, lipomas, or LSTs, thus preventing unnecessary interventions. Furthermore, adherence to strict disinfection protocols and careful endoscopic techniques is vital in minimizing the occurrence of iatrogenic CP. Continued awareness and research into CP's pathogenesis and potential complications are essential for improving diagnostic accuracy and patient management.

## References

[1] Iwamuro M, Tanaka T, Kawabata T, Sugihara Y, Harada K, Hiraoka S, Okada H (2018). Pseudolipomatosis of the colon and cecum followed by pneumatosis intestinalis. Intern Med.

[2] Bseiso B, Hussain I, Almomen S, AlGhamdi SM, Alabdrabnabi F, Al-Salem AH (2017). Colonic mucosal pseudolipomatosis: diagnosis and etiology. Adv Res Gastroentero Hepatol.

[3] Kaassis M, Croue A, Carpentier S, Burtin P, Boyer J (1997). A case of colonic pseudolipomatosis: a rare complication of colonoscopy?. Endoscopy.

[4] Brevet M, Chatelain D, Bartoli E (2006). Colonic pseudolipomatosis: clinical, endoscopical and pathological features in nine cases. Gastroenterol Clin Biol.

[5] Iwamuro M, Tanaka T, Yamauchi N (2020). Cytomegalovirus colitis followed by colonic pseudolipomatosis and gastric emphysema in a post-resuscitation patient. Intern Med.

[6] Martinez CAR, Souza CAF, Noronha MR, Alfredo CH, Spadari APP, Bartocci PCM, Priolli DG (2008). Pseudolipomatosis of the colon: a case report. Rev Bras.

[7] Silva SM, Coura MM, Seidler HB, Silva SM (2022). Colonic pseudolipomatosis: a rare but characteristic endoscopic condition. Am J Case Rep.

[8] Nivatvongs S (1986). Complications in colonoscopic polypectomy. An experience with 1,555 polypectomies. Dis Colon Rectum.

[9] Nakasono M, Hirokawa M, Muguruma N (2006). Colonic pseudolipomatosis, microscopically classified into two groups. J Gastroenterol Hepatol.

[10] Waring JP, Manne RK, Wadas DD, Sanowski RA (1989). Mucosal pseudolipomatosis: an air pressure-related colonoscopy complication. Gastrointest Endosc.

